# Prevalence and clinical characteristics of metabolically healthy obese individuals and other obese/non-obese metabolic phenotypes in a working population: results from the Icaria study

**DOI:** 10.1186/s12889-016-2921-4

**Published:** 2016-04-01

**Authors:** Albert Goday, Eva Calvo, Luis Alberto Vázquez, Elena Caveda, Teresa Margallo, Carlos Catalina-Romero, Jesús Reviriego

**Affiliations:** Servicio de Endocrinología y Nutrición, Hospital del Mar and Departament de Medicia, Universitat Autonoma Barcelona, CIBERobn, ISCIII, Barcelona, Spain; Ibermutuamur, Mutua de Accidentes de Trabajo y Enfermedades Profesionales de la Seguridad Social n° 274, Madrid, Spain; Eli Lilly and Company, Alcobendas, Spain; Sociedad de Prevención de Ibermutuamur (Ibermutuamur Prevention Society), Madrid, Spain

**Keywords:** Metabolically healthy obesity, Metabolic risk factors, Prevalence, Working population

## Abstract

**Background:**

Metabolically healthy obese (MHO) phenotype may present with distinct characteristics compared with those with a metabolically unhealthy obese phenotype. Epidemiologic data on the distribution of these conditions in the working population are lacking. We aimed to evaluate the prevalence and clinical characteristics of MHO and other obese/non-obese metabolic phenotypes in a working population.

**Methods:**

Cross-sectional analysis of all subjects who had undergone a medical examination with Ibermutuamur Prevention Society from May 2004 to December 2007. Participants were classified into 5 categories according to their body mass index (BMI); within each of these categories, participants were further classified as metabolically healthy (MH) or metabolically unhealthy (MUH) according to the modified NCEP-ATPIII criteria. A logistic regression analysis was performed to evaluate some clinically relevant factors associated with a MH status.

**Results:**

In the overall population, the prevalence of the MHO phenotype was 8.6 %. The proportions of MH individuals in the overweight and obese categories were: 87.1 % (overweight) and 55.5 % (obese I-III [58.8, 40.0, and 38.7 % of the obese I, II, and III categories, respectively]). When the overweight and obese categories were considered, compared with individuals who were MUH, those who were MH tended to be younger and more likely to be female or participate in physical exercise; they were also less likely to smoke, or to be a heavy drinker. In the underweight and normal weight categories, compared with individuals who were MH, those who were MUH were more likely to be older, male, manual (blue collar) workers, smokers and heavy drinkers. Among participants in the MUH, normal weight group, the proportion of individuals with a sedentary lifestyle was higher relative to those in the MH, normal weight group. The factors more strongly associated with the MUH phenotype were BMI and age, followed by the presence of hypercholesterolemia, male sex, being a smoker, being a heavy drinker, and lack of physical exercise.

**Conclusions:**

The prevalence of individuals with a MHO phenotype in the working population is high. This population may constitute an appropriate target group in whom to implement lifestyle modification initiatives to reduce the likelihood of transition to a MUH phenotype.

## Background

Obesity is a major public health problem. It affects more than 1.7 billion people and is the sixth most important risk factor contributing to the overall burden of disease worldwide [1]. Obesity and overweight have been associated with an increased risk of developing type 2 diabetes, dyslipidemia, hypertension, coronary heart disease, stroke, and cancer, among many other diseases [1–3], and these conditions are associated with a reduced life expectancy [4].

Among obese individuals, a phenotype of patients who do not present metabolic abnormalities, the so-called metabolically healthy obese (MHO) phenotype, has been described [5]. The prevalence of the MHO phenotype varies greatly across studies (2.2–11.9 % of the general population and 6–40 % of the obese population), depending on the study design and, particularly, on the criteria used for its definition [5]. Individuals with this phenotype could be at a lower risk of developing the aforementioned health problems compared with metabolically unhealthy obese (MUHO) individuals. However, recent investigations have shown that the MHO phenotype is associated with subclinical cardiovascular markers, an increased risk of developing diabetes, and even an increased risk of all-cause mortality and/or cardiovascular events in the long term [6–9].

MHO individuals may present with distinct characteristics compared with MUHO individuals. Phillips et al. characterize the former as having less disturbed coordination of the pathways involved in nutrient handling, insulin signaling, inflammation, and lipid metabolism, which may make them more responsive to dietary interventions [5]. Consequently, it may be important to identify individuals belonging to a MHO or MUHO phenotype to aid selection of the appropriate therapeutic intervention [10]. In this context, the working population may be an appropriate group in whom to apply this management approach. However, to date, there have not been any studies evaluating MHO individuals in the working population.

The aim of this study was to evaluate the prevalence of the MHO and other obese/non-obese metabolic phenotypes and their clinical characteristics in a working population.

## Methods

### Study design and population

This cross-sectional analysis was part of the Ibermutuamur CArdiovascular RIsk Assessment (ICARIA) study. The methodology of the ICARIA study has been described elsewhere [11].

Briefly, the ICARIA project included workers whose companies have healthcare coverage with Ibermutuamur, a single nationwide Spanish workers’ compensation insurance company that covers 8 % of the Spanish working population and includes workers from all activity sectors and all geographical areas of Spain [12]. To be included in these analyses, participants had to have undergone a routine medical examination with Ibermutuamur Prevention Society between May 2004 and December 2007, and they had to have information available regarding all variables included in the definition of “metabolically healthy” (see later).

### Evaluations

The routine medical check-ups included a structured questionnaire, a physical examination and a laboratory assessment.

The structured questionnaire included information on age, sex, specific occupation, tobacco and alcohol consumption, physical exercise, and medical history. Regarding occupation, participants were categorized as either manual (blue-collar) workers or non-manual (white-collar) workers [13]. Smoking status was categorized as never smoker, former smoker (stopped smoking ≥1 year ago), former smoker (stopped smoking <1 year ago), and current smoker. Alcohol consumption was categorized as high if they consumed 14 or more standard drinks per week; the following conversion guide was used: 1 glass of wine = 1 drink, 1 beer = 1 drink, 1 glass of spirit or mix spirit = 2 drinks. Individuals’ physical exercise level was categorized into four groups according to self-reported information: no physical exercise, <2 h/week of physical exercise, ≥2 h/week of physical exercise, and regular physical exercise.

The physical examination included weight, height, waist circumference, and two blood pressure recordings from the same arm. Waist circumference was measured at the midpoint between the lowest rib and the iliac crest. Blood pressure was measured using a validated automatic measuring system (OMRON M4-1, Omron Electronics, Hoofddorp, Netherlands). Laboratory assays included fasting serum glucose, triglycerides, total cholesterol, and high-density lipoprotein (HDL) cholesterol.

### Ethical issues

The study was reviewed and approved by the Ethics Committee of Ibermutuamur (Madrid, Spain). All participants provided consent to include their information in anonymous aggregated analyses for the ICARIA project. The study was conducted according to the principles of the Declaration of Helsinki.

### Statistical analysis

According to body mass index (BMI), participants were categorized as underweight (BMI: <18.5 kg/m^2^), normal weight (BMI: 18.5–24.99 kg/m^2^), overweight (BMI: 25.0–29.99 kg/m^2^) or obese (BMI: ≥30 kg/m^2^). Obese individuals were further categorized as obese I (BMI: 30.0–34.99 kg/m^2^), obese II (BMI: 35.0–39.99 kg/m^2^) or obese III (BMI: ≥40 kg/m^2^). Metabolic health was evaluated using the modified criteria for metabolic syndrome according to the National Cholesterol Education Program (NCEP) Adult Treatment Panel III (ATPIII) guidelines as in previous studies [14]: waist circumference >102 cm (>40 in) for men or >88 cm (>35 in) for women, triglycerides ≥150 mg/dL or receiving treatment for hyperlipidemia, HDL cholesterol <40 mg/dL for men or <50 mg/dL for women, blood pressure ≥130/85 mmHg or previous diagnosis of hypertension or receiving treatment for hypertension, fasting glucose ≥100 mg/dL or receiving treatment for diabetes. Combined BMI and metabolic health phenotypes were defined based on BMI category and whether individuals met 0 to 2 (metabolically healthy) or 3 or more (metabolically unhealthy) NCEP-ATPIII criteria. As part of an exploratory analysis, we also used a more stringent criterion for MHO, that is, obese individuals who did not meet any criteria of the NCEP-ATPIII guideline.

Continuous outcomes are presented as means (standard deviations [SD]), and categorical outcomes are presented as relative frequencies. We also calculated the 95 % confidence intervals (CIs) for all parameters. Differences among BMI with metabolically healthy or unhealthy phenotypes were tested. Chi-squared test was used for categorical variables. Student *t*-test and one-way ANOVA were selected to compare groups in quantitative variables.

To evaluate the factors associated with a metabolically healthy status, a logistic regression analysis was performed using metabolic unhealthy status as the dependent variable and age categories (≤34, 35–44, 45–54, and ≥55 years), sex, BMI categories (as described earlier), type of worker, smoking status, alcohol consumption, presence of hypercholesterolemia and physical exercise as the explanatory variables.

All analyses were performed using SPSS version 17 (SPSS Inc., Chicago, IL, USA).

## Results

During the study period, 451,432 individuals were assessed and provided data for all of the parameters used to define metabolic health status.

### Prevalence of BMI and metabolic phenotypes

The prevalences of the different BMI categories were as follows: underweight (1.7 %), normal weight (44.8 %), overweight (38.0 %), obese I (12.5 %), obese II (2.4 %) and obese III (0.6 %). Overall, 70,053 individuals (15.5 %) were obese.

The prevalences of each combination of BMI and metabolic phenotype in the total study population are presented in Tables 1, 2 and 3, with overall values of 8.6 % for MHO and 7.0 % for MUHO.

**Table 1:** Characteristics of the study population by metabolic phenotype among overweight and obese individuals

Characteristics	Overweight (BMI^a^ 25.00–29.99 kg/m^2^)		Obese All (BMI^a^ ≥30.00 kg/m^2^)		p-value^*^	
	*n* = 171,368		*n* = 70,052			
	Healthy	Unhealthy	Healthy	Unhealthy	Healthy	Unhealthy
	*n* = 149,231	*n* = 22,137	*n* = 38,600	*n* = 31,452		
Prevalence, %	33.1	4.9	8.6	7.0		
(95 % CI) ^b^	(32.9–33.2)	(4.8–5.0)	(8.5–8.6)	(6.9–7.0)		
Age (years), mean [SD]^c^ (95 % CI)^b^	37.3 [10.5]	44.1 [10.6]	37.7 [10.7]	42.9 [10.7]	<0.0001	<0.0001
	(37.2–37.4)	(44.0–44.3)	(37.6–37.8)	(42.7–43.0)		
Female, %	16.5	11.7	18.4	11.9	<0.0001	0.5889
(95 % CI)^b^	(16.3–16.7)	(11.3–12.1)	(18.0–18.8)	(11.5–12.2)		
Blue-collar worker, %	69.8	71.3	74.2	75.2	<0.0001	<0.0001
(95 % CI)^b^	(69.6–70.0)	(70.7–71.9)	(73.7–74.6)	(74.7–75.6)		
Smoking status, % (95 % CI)^b^						
Never smoker	38.9	29.5	38.6	30.1	<0.0001	<0.0001
	(38.6–39.1)	(28.9–30.1)	(38.1–39.1)	(29.6–30.6)		
Former smoker (stopped smoking ≥1 year ago)	15.2	20.3	16.8	21.4		
	(15.0–15.4)	(19.7–20.8)	(16.4–17.2)	(20.9–21.8)		
Former smoker (stopped smoking <1 year ago)	3.2	3.5	3.3	3.7		
	(3.1–3.3)	(3.3–3.8)	(3.2–3.5)	(3.5–4.0)		
Current smoker	42.7	46.8	41.3	44.8		
	(42.5–43.0)	(46.1–47.4)	(40.8–41.8)	(44.2–45.3)		
Heavy drinker, %	2.1	3.0	2.1	3.3	0.8921	0.078
(95 % CI)^b^	(2.1–2.2)	(2.8–3.3)	(2.0–2.3)	(3.1–3.5)		
Physical exercise^d^, % (95 % CI)^b^						
Regular physical exercise	21.5	14.6	15.9	11.4	<0.0001	<0.0001
	(21.0–21.9)	(13.5–15.8)	(15.2–16.6)	(10.7–12.3)		
≥2 h/week of physical exercise	10.2	10.2	10.7	9.0		
	(9.9–10.6)	(9.2–11.2)	(10.1–11.3)	(8.3–9.8)		
<2 h/week physical exercise	16.5	15.3	14.8	13.0		
	(16.1–16.9)	(14.2–16.5)	(14.2–15.5)	(12.2–13.9)		
No physical exercise	51.8	59.9	58.6	66.5		
	(51.3–52.3)	(58.3–61.5)	(57.6–59.5)	(65.3–67.7)		
Systolic blood pressure (mmHg), mean [SD]^c^ (95 % CI)^b^	127.3 [15.2]	139.9 [16.0]	130.4 [15.9]	141.6 [16.8]	<0.0001	<0.0001
	(127.2–127.4)	(139.6–140.1)	(130.2–130.6)	(141.5–141.8)		
Diastolic blood pressure (mmHg), mean [SD]^c^ (95 % CI)^b^	76.8 [10.1]	84.3 [10.2]	79.6 [10.6]	86.2 [10.8]	<0.0001	<0.0001
	(76.8–76.9)	(84.1–84.4)	(79.5–79.7)	(86.1–86.3)		
Hypertension, %	44.6	89.6	52.2	89.1	<0.0001	0.0810
(95 % CI)^b^	(44.4–44.9)	(89.2–90.0)	(51.7–52.7)	(88.8–89.5)		
HDL cholesterol (mg/dL), mean [SD]^c^ (95 % CI)^b^	51.4 [12.0]	43.1 [11.3]	50.4 [11.3]	43.8 [10.7]	<0.0001	<0.0001
	(51.3–51.4)	(42.9–43.2)	(50.3–50.5)	(43.6–43.9)		
Triglycerides (mg/dL), mean [SD]^c^ (95 % CI)^b^	109.2 [70.0]	210.1 [133.4]	114.2 [69.1]	198.1 [127.7]	<0.0001	<0.0001
	(108.9–109.6)	(208.3–211.8)	(113.5–114.9)	(196.7–199.5)		
Hypertriglyceridemia, %	17.3	77.9	15.5	66.9	<0.0001	<0.0001
(95 % CI)^b^	(17.1–17.5)	(77.3–78.4)	(15.2–15.9)	(66.4–67.4)		
Total cholesterol (mg/dL), mean [SD]^c^ (95 % CI)^b^	201.4 [40.5]	217.5 [44.0]	203.9 [38.6]	216.1 [42.6]	<0.0001	0.0003
	(201.2–201.6)	(216.9–218.1)	(203.5–204.3)	(215.6–216.6)		
Hypercholesterolemia, %	50.2	69.0	53.0	66.9	<0.0001	<0.0001
(95 % CI)^b^	(49.9–50.4)	(68.4–69.6)	(52.5–53.5)	(66.4–67.5)		
Glucose level (mg/dL), mean [SD]^c^ (95 % CI)	89.2 [14.7]	107.4 [33.0]	89.5 [12.9]	106.7 [33.3]	0.0008	0.0092
	(89.1–89.3)	(107.0–107.9)	(89.3–89.6)	(106.3–107)		
BMI (kg/m^2^), mean [SD]^c^ (95 % CI)^b^	27.1 [1.4]	27.8 [1.4]	32.6 [3.0]	33.7 [3.4]	<0.0001	<0.0001
	(27.1–27.1)	(27.8–27.9)	(32.5–32.6)	(33.7–33.7)		
Waist circumference (cm), mean [SD]^c^ (95 % CI)^b^	91.1 [7.5]	97.6 [7.5]	102.1 [9.7]	109.2 [9.0]	<0.0001	<0.0001
	(91.1–91.2)	(97.5–97.7)	(102–102.2)	(109.1–109.3)		

^*^P-values correspond to chi-squared test for categorical data, and *t*-test for continuous variables

^a^BMI: body mass index

^b^95% CI: 95 % confidence interval

^c^SD: standard deviation

^d^Information on physical exercise was only available for a total of 100,561 study participants

**Table 2:** Characteristics of the study population by metabolic phenotype among obese I, II and III individuals

Characteristics	Obese I (BMI^a^ 30.00–34.99 kg/m^2^)		Obese II (BMI^a^ 35.00–39.99 kg/m^2^)		Obese III (BMI^a^ ≥40.00 kg/m^2^)		p-value^*^	
	*n* = 56,478		*n* = 10,878		*n* = 2,696			
	Healthy	Unhealthy	Healthy	Unhealthy	Healthy	Unhealthy	Healthy	Unhealthy
	*n* = 33,205	*n* = 23,273	*n* = 4,352	*n* = 6,526	*n* = 1,043	*n* = 1,653		
Prevalence, %	7.4	5.2	1.0	1.4	0.2	0.4		
(95 % CI)^b^	(7.3–7.4)	(5.1–5.2)	(0.9–1.0)	(1.4–1.5)	(0.2–0.2)	(0.3–0.4)		
Age (years), mean [SD] ^c^ (95 % CI)^b^	37.9 [10.7]	43.5 [10.6]	36.6 [10.6]	41.5 [10.8]	35.8 [9.9]	39.8 [10.3]	<0.0001	<0.0001
	(37.8–38.0)	(43.3–43.6)	(36.2–36.9)	(41.2–41.7)	(35.2–36.4)	(39.3–40.3)		
Female, %	16.6	10.5	28.2	14.1	34.2	22.3	<0.0001	<0.0001
(95 % CI)^b^	(16.2–17.0)	(10.1–10.9)	(26.9–29.6)	(13.3–14.9)	(31.3–37.1)	(20.4–24.4)		
Blue-collar worker, %	74.1	74.6	75.0	77.4	72.3	74.1	0.1984	<0.0001
(95 % CI)^b^	(73.7–74.6)	(74–75.2)	(73.7–76.3)	(76.4–78.4)	(69.5–75)	(71.9–76.1)		
Smoking status, % (95 % CI)^b^								
Never smoker	38.4	29.7	39.1	31.1	42.0	32.3	0.0097	<0.0001
	(37.9–38.9)	(29.1–30.3)	(37.7–40.6)	(29.9–32.2)	(39.0–45.0)	(30.1–34.6)		
Former smoker (stopped smoking ≥1 year ago)	17.0	22.3	15.8	18.6	13.6	18.6		
	(16.6–17.4)	(21.8–22.9)	(14.7–16.9)	(17.7–19.6)	(11.7–15.8)	(16.8–20.6)		
Former smoker (stopped smoking <1 year ago)	3.4	3.7	3.1	3.7	2.7	3.8		
	(3.2–3.6)	(3.5–4.0)	(2.7–3.7)	(3.3–4.2)	(1.9–3.9)	(2.9–4.8)		
Current smoker	41.2	44.2	42.0	46.6	41.7	45.3		
	(40.7–41.7)	(43.6–44.9)	(40.5–43.4)	(45.4–47.8)	(38.8–44.7)	(42.9–47.7)		
Heavy drinker, %	2.2	3.3	2.0	3.4	2.5	2.8	0.6390	0.5314
(95 % CI)^b^	(2.0–2.3)	(3.1–3.6)	(1.6–2.5)	(3.0–3.8)	(1.7–3.6)	(2.1–3.8)		
Physical exercise^d^, % (95 % CI)^b^								
Regular physical exercise	16.3	12.0	14.0	10.2	14.4	9.0	0.0012	0.1334
	(15.5–17.1)	(11.1–13.0)	(12.3–16.0)	(8.7–11.9)	(11.1–18.5)	(6.6–12.3)		
≥2 h/week of physical exercise	10.9	9.1	9.8	9.3	8.6	7.2		
	(10.3–11.6)	(8.3–10.0)	(8.3–11.5)	(7.9–10.9)	(6.1–12.1)	(5.1–10.3)		
<2 h/week physical exercise	15.1	13.3	14.4	12.3	9.8	12.9		
	(14.4–15.9)	(12.3–14.3)	(12.6–16.3)	(10.7–14.2)	(7.1–13.4)	(9.9–16.6)		
No physical exercise	57.7	65.6	61.8	68.2	67.1	70.8		
	(56.7–58.8)	(64.2–67.0)	(59.1–64.4)	(65.7–70.6)	(62–71.9)	(66.1–75.1)		
Systolic blood pressure (mmHg), mean [SD]^c^ (95 % CI)^b^	130.2 [15.8]	141.2 [16.6]	131.2 [16.5]	142.7 [17.3]	132.2 [17.8]	143.8 [17.1]	<0.0001	<0.0001
	(130.1–130.4)	(141–141.4)	(130.7–131.7)	(142.3–143.2)	(131.1–133.2)	(143–144.6)		
Diastolic blood pressure (mmHg), mean [SD]^c^ (95 % CI)^b^	79.4 [10.5]	85.8 [10.6]	80.6 [10.9]	87.1 [11.1]	81.8 [12.3]	88.1 [11.4]	<0.0001	<0.0001
	(79.3–79.5)	(85.6–85.9)	(80.3–81.0)	(86.9–87.4)	(81.1–82.5)	(87.5–88.6)		
Hypertension, %	52.1	88.6	52.5	90.4	56.0	90.9	0.036	<0.0001
(95 % CI)^b^	(51.5–52.6)	(88.2–89.0)	(51.0–54.0)	(89.7–91.1)	(53.0–59.0)	(89.5–92.2)		
HDL cholesterol (mg/dL), mean [SD]^c^ (95 % CI)^b^	50.3 [11.3]	43.8 [10.7]	51.1 [11.3]	43.4 [10.4]	51.3 [11.4]	43.8 [10.6]	<0.0001	0.0257
	(50.2–50.4)	(43.7–44.0)	(50.8–51.4)	(43.2–43.7)	(50.6–52.0)	(43.2–44.3)		
Triglycerides (mg/dL), mean [SD]^c^ (95 % CI)^b^	115.7 [71.7]	200.0 [130.0]	105.7 [49.2]	194.3 [121.8]	103.8 [50.2]	186.2 [116.6]	<0.0001	<0.0001
	(114.9–116.4)	(198.3–201.6)	(104.2–107.2)	(191.4–197.3)	(100.6–106.7)	(180.6–191.9)		
Hypertriglyceridemia, %	16.7	68.1	8.8	64.6	6.8	59.0	<0.0001	<0.0001
(95 % CI)^b^	(16.3–17.1)	(67.5–68.7)	(8.0–9.7)	(63.4–65.8)	(5.4–8.5)	(56.6–61.4)		
Total cholesterol (mg/dL), mean [SD]^c^ (95 % CI)^b^	204.6 [38.7]	217.4 [42.7]	199.9 [37.6]	213.5 [42.2]	196.4 [35.2]	208.4 [40.5]	<0.0001	<0.0001
	(204.2–205.0)	(216.8–217.9)	(198.8–201)	(212.5–214.5)	(194.3–198.6)	(206.5–210.4)		
Hypercholesterolemia, %	53.8	68.3	48.8	64.2	45.4	58.7	<0.0001	<0.0001
(95 % CI)^b^	(53.2–54.3)	(67.7–68.9)	(47.3–50.3)	(63.0–65.4)	(42.4–48.4)	(56.3–61.0)		
Glucose level (mg/dL), mean [SD]^c^ (95 % CI)	89.6 [13.1]	106.3 [32.6]	88.5 [11.5]	107.2 [34.8]	87.7 [10.0]	109.4 [37.3]	<0.0001	0.0021
	(89.5–89.8)	(105.9–106.8)	(88.2–88.9)	(106.3–108.0)	(87.1–88.3)	(107.6–111.2)		
BMI (kg/m^2^), mean [SD]^c^ (95 % CI)^b^	31.7 [1.3]	32.1 [1.4]	36.8 [1.3]	36.9 [1.4]	44.2 [5.7]	43.3 [4.3]	<0.0001	<0.0001
	(31.6–31.7)	(32.1–32.1)	(36.7–36.8)	(36.8–36.9)	(43.9–44.7)	(43.2–43.7)		
Waist circumference (cm), mean [SD]^c^ (95 % CI)^b^	100.6 [8.2]	106.4 [6.8]	110.1 [10.9]	115.1 [8.2]	116.3 [16.8]	125 [10.7]	<0.0001	<0.0001
	(100.5–100.7)	(106.3–106.5)	(109.8–110.4)	(114.9–115.2)	(115.2–117.3)	(124.5–125.5)		

^*^ P-values correspond to chi-squared test for categorical data, and one-way ANOVA for continuous variables

^a^BMI: body mass index

^b^95% CI: 95 % confidence interval

^c^SD: standard deviation

^d^Information on physical exercise was only available for a total of 100,561 study participants

**Table 3:** Characteristics of the study population by metabolic phenotype among underweight and normal weight individuals

Characteristics	Underweight (BMI^a^ <18.50 kg/m^2^)		Normal weight (BMI^a^ 18.50–24.99 kg/m^2^)		p-value^*^	
	*n* = 7,747		*n* = 202,265			
	Healthy	Unhealthy	Healthy	Unhealthy	Healthy	Unhealthy
	*n* = 7,711	*n* = 36	*n* = 197,846	*n* = 4,419		
Prevalence, %	1.7	0.0	43.8	1.0		
(95 % CI)^b^	(1.7–1.7)	(0.0–0.0)	(43.7–44)	(1.0–1.0)		
Age (years), mean [SD]^c^ (95 % CI)^b^	28.4 [8.3]	38.6 [12.4]	32.8 [9.7]	41.8 [11.4]	<0.0001	0.0988
	(28.2–28.6)	(34.4–42.8)	(32.7–32.8)	(41.4–42.1)		
Female, %	66.2	16.7	40.7	16.2	<0.0001	0.9403
(95 % CI)^b^	(65.2–67.3)	(7.9–31.9)	(40.4–40.9)	(15.1–17.3)		
Blue-collar worker, %	54.7	66.7	60.4	72.8	<0.0001	0.4176
(95 % CI)^b^	(53.5–55.8)	(50.3–79.8)	(60.2–60.7)	(71.5–74.1)		
Smoking status, % (95 % CI)^b^						
Never smoker	37.6	11.1	39.3	26.3	<0.0001	0.0570
	(36.5–38.7)	(4.4–25.3)	(39.1–39.6)	(25–27.6)		
Former smoker (stopped smoking ≥1 year ago)	5.6	16.7	9.4	13.6		
	(5.1–6.1)	(7.9–31.9)	(9.3–9.6)	(12.6–14.6)		
Former smoker (stopped smoking <1 year ago)	2.4	0.0	2.7	2.8		
	(2.1–2.8)	(0.0–9.6)	(2.6–2.7)	(2.4–3.3)		
Current smoker	54.5	72.2	48.6	57.4		
	(53.4–55.6)	(56.0–84.2)	(48.3–48.8)	(55.9–58.8)		
Heavy drinker, %	1.3	2.8	1.8	3.5	0.0001	0.8113
(95 % CI)^b^	(1.0–1.5)	(0.5–14.2)	(1.8–1.9)	(3.0–4.1)		
Physical exercise^d^, % (95 % CI)^b^						
Regular physical exercise	16.6	0.0	23.1	16.2	<0.0001	0.5628
	(15.1–18.2)	(0.0–43.4)	(22.7–23.5)	(13.6–19.2)		
≥2 h/week of physical exercise	9.1	20.0	9.7	10.9		
	(8.0–10.4)	(3.6–62.4)	(9.5–10.0)	(8.7–13.4)		
<2 h/week physical exercise	11.9	20.0	16.0	13.7		
	(10.6–13.4)	(3.6–62.4)	(15.7–16.4)	(11.3–16.5)		
No physical exercise	62.3	60.0	51.1	59.2		
	(60.3–64.4)	(23.1–88.2)	(50.7–51.6)	(55.5–62.9)		
Systolic blood pressure (mmHg), mean [SD]^c^ (95 % CI)^b^	112.5 [13.5]	136.7 [14.9]	119.8 [14.5]	138.1 [15.3]	<0.0001	0.5729
	(112.2–112.8)	(131.6–141.7)	(119.8–119.9)	(137.6–138.5)		
Diastolic blood pressure (mmHg), mean [SD]^c^ (95 % CI)^b^	69.4 [9.3]	83.3 [11.2]	72.2 [9.5]	82.3 [10.3]	<0.0001	0.5457
	(69.2–69.6)	(79.5–87.1)	(72.2–72.3)	(82.0–82.6)		
Hypertension, %	12.9	86.1	26.2	90.7	<0.0001	0.3732
(95 % CI)^b^	(12.2–13.7)	(71.3–93.9)	(26.0–26.4)	(89.8–91.5)		
HDL cholesterol (mg/dL), mean [SD]^c^ (95 % CI)^b^	60.4 [14.7]	43.4 [14.4]	56.5 [13.9]	43.5 [12.4]	<0.0001	0.9765
	(60.1–60.8)	(38.6–48.3)	(56.5–56.6)	(43.1–43.9)		
Triglycerides (mg/dL), mean [SD]^c^ (95 % CI)^b^	68.5 [31.1]	226.4 [246.2]	82.5 [50.2]	209.2 [133.7]	<0.0001	0.6787
	(67.8–69.2)	(143.1–309.7)	(82.3–82.7)	(205.3–213.2)		
Hypertriglyceridemia, %	2.4	75.0	7.0	83.1	<0.0001	0.2215
(95 % CI)^b^	(2.1–2.8)	(58.9–86.2)	(6.9–7.2)	(82.0–84.2)		
Total cholesterol (mg/dL), mean [SD]^c^ (95 % CI)^b^	172.3 [33.7]	194.7 [51.4]	185.6 [37.4]	209.8 [45.5]	<0.0001	0.0480
	(171.6–173.1)	(177.3–212.1)	(185.4–185.8)	(208.4–211.1)		
Hypercholesterolemia, %	19.8	47.2	33.1	61.0	<0.0001	0.0965
(95 % CI)^b^	(18.9–20.7)	(32.0–63.0)	(32.9–33.3)	(59.5–62.4)		
Glucose level (mg/dL), mean [SD]^c^ (95 % CI)^b^	82.6 [12.5]	105.4 [38.5]	85.6 [13.9]	108.3 [38.7]	<0.0001	0.6625
	(82.3–82.9)	(92.4–118.4)	(85.5–85.6)	(107.1–109.4)		
BMI (kg/m^2^), mean [SD]^c^ (95 % CI)^b^	17.7 [0.7]	16.8 [1.6]	22.4 [1.7]	23.5 [1.3]	<0.0001	<0.0001
	(17.7–17.7)	(16.3–17.3)	(22.4–22.4)	(23.4–23.5)		
Waist circumference (cm), mean [SD]^c^ (95 % CI)^b^	67.9 [6.4]	84.4 [17.5]	78.6 [8.1]	86.8 [8.3]	<0.0001	0.4090
	(67.7–68.0)	(78.4–90.3)	(78.6–78.6)	(86.6–87.1)		

^*^ P-values correspond to chi-squared test for categorical data, and *t*-test for continuous variables

^a^BMI: body mass index

^b^95% CI: 95 % confidence interval

^c^SD: standard deviation

^d^Information on physical exercise was only available for a total of 100,561 study participants

**Fig. 1:**
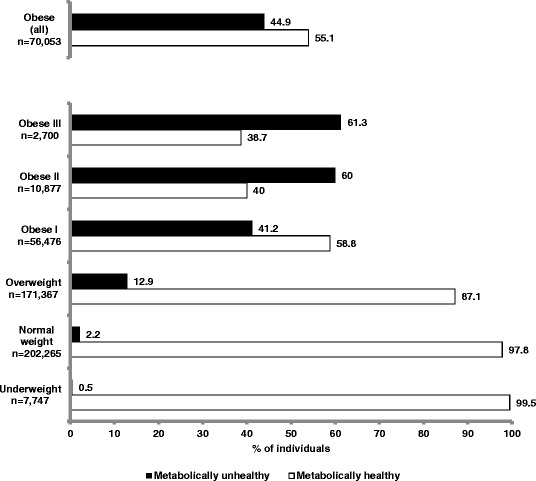
Prevalence of metabolically healthy/unhealthy individuals in the different BMI categories

The prevalence of a metabolically healthy status was 87.1 % for the overweight individuals and 55.1 % for obese individuals. Among the various obesity categories, the prevalence of metabolically healthy individuals was 58.8 % for obesity I, 40.0 % for obesity II, 38.7 % for obesity III (Fig. 1). Using the more stringent criteria for defining metabolically healthy status (none of the 5 NCEP-ATPIII criteria), there were no individuals with a metabolically healthy phenotype among the obese II and III categories, and the proportion of metabolically healthy individuals among obese I and overweight subjects was 6.4 and 25.7 %, respectively (data not shown).

Among individuals who were underweight or normal weight, 99.5 and 97.8 %, respectively, were metabolically healthy using the modified NCEP-ATPIII criteria. Using the more stringent criteria, proportions were 70.6 and 53.6 %, respectively (data not shown).

### Characteristics associated with a metabolically healthy phenotype among overweight and obese individuals

When the overweight and obese categories were considered, compared with individuals with metabolically unhealthy phenotypes, individuals who were metabolically healthy tended to be younger and more likely to be female or participate in physical exercise; they were also less likely to smoke or to be a heavy drinker (Table 1). Overall, the proportions of blue-collar workers were similar between healthy and unhealthy phenotypes for the overweight (69.8 % versus 71.3 %) and obese categories (74.2 % versus 75.2 %).

In MHO individuals, mean values of systolic blood pressure, diastolic blood pressure, HDL cholesterol, and glucose showed less than 3 % variation among the three categories of obesity (Table 2). This degree of variation among groups was statistically significant (p < 0.0001 for all), but was not deemed clinically relevant. In contrast, mean triglyceride levels were 115.7 mg/dL, 105.7 mg/dL and 103.7 mg/dL for these individuals in the obese I, II, and III categories (p < 0.0001), respectively. Similarly, mean total cholesterol levels were 204.6 mg/dL in obese I, 199.9 mg/dL in obese II, and 196.4 mg/dL in obese III metabolically healthy individuals (p < 0.0001). Respective mean waist circumferences were 100.6 cm, 110.1 cm, and 116.2 cm (p < 0.0001).

Total cholesterol values were lower in individuals with the metabolically healthy phenotype compared with those with the metabolically unhealthy phenotype in both the overweight (201.4 vs. 217.5 mg/dL) and obese (203.9 vs. 216.1 mg/dL) groups (Table 1).

### Characteristics associated with a metabolically unhealthy phenotype among underweight and normal weight individuals

In the underweight or normal weight categories, compared with metabolically healthy individuals, those who were metabolically unhealthy were more likely to be older, male, blue-collar workers, smokers, and heavy drinkers (Table 3). Within the metabolically unhealthy normal weight phenotype group, the proportion of individuals with a sedentary lifestyle was higher relative to that in the metabolically healthy normal weight phenotype group.

There were no notable differences in metabolic risk factors between metabolically unhealthy individuals who were underweight or normal weight, with the exceptions of mean triglyceride levels, which were higher among underweight individuals compared with normal weight individuals (226.4 vs. 209.2 mg/dL), and total cholesterol levels, which were lower among underweight individuals (194.7 vs. 209.8 mg/dL); however, only the differences in total cholesterol levels were statistically significant (*p* = 0.0480) (Table 3).

In the underweight category, mean triglyceride levels of individuals in the metabolically unhealthy phenotype were 226.4 mg/dL; for individuals in the metabolically healthy phenotype, they were 68.5 mg/dL. The greatest difference in mean waist circumference between metabolically unhealthy and healthy phenotypes was observed among underweight individuals (84.4 versus 67.9 cm). In the normal weight category, mean triglyceride levels were 209.2 mg/dL in metabolically unhealthy individuals and 82.5 mg/dL in metabolically healthy individuals. Among underweight or normal weight individuals who were metabolically unhealthy, mean total cholesterol levels were 194.7 and 209.8 mg/dL, respectively, and underweight or normal weight individuals who were metabolically healthy had mean total cholesterol levels of 172.3 and 185.6 mg/dL, respectively.

### Factors associated with the metabolic unhealthy phenotype

In a regression model, the factors most strongly associated with the metabolic unhealthy phenotype were BMI and age (Table 4). Individuals who were underweight had a lower likelihood of having a metabolic unhealthy phenotype compared with those with normal weight (adjusted odds ratio [aOR] 0.25; 95 % CI 0.11–0.62). Obese individuals had a marked increase in the likelihood of exhibiting a metabolic unhealthy phenotype compared with normal weight individuals, ranging from an aOR of 24 (95 % CI, 22–26) among those in the obese I category to an aOR of 67 (95 % CI, 56–80) among individuals in the obese III category. Increased age was associated with increased likelihood of expressing a metabolically unhealthy phenotype (35–44 years versus ≤34 years: aOR 1.94, 95 % CI 1.83–2.07; 45–54 years versus ≤34 years: aOR 3.18, 95 % CI 2.97–3.40; ≥55 years versus ≤34 years: aOR 4.95, 95 % CI 4.55–5.39). There was almost a five-fold increase in risk among subjects aged 55 years or older compared with those aged ≤34 years. Other factors associated with the metabolically unhealthy phenotype were the presence of hypercholesterolemia, male sex, smoking, heavy drinking, and no physical exercise.

**Table 4:** Factors associated with expression of a metabolically unhealthy phenotype (unadjusted and multivariate analyses)

Factor	Crude OR^a^	p-value	Adjusted OR^a^	p-value
	95 % CI^b^		95 % CI^b^	
Age (years)		<0.0001		<0.0001
≤34	1.00	-	1.00	-
35–44	2.67	<0.0001	1.94	<0.0001
	(2.61–2.73)		(1.83–2.07)	
45–54	5.01	<0.0001	3.18	<0.0001
	(4.89–5.14)		(2.97–3.40)	
55	7.72	<0.0001	4.95	<0.0001
	(7.50–7.95)		(4.55–5.39)	
Male [Female]	3.07	<0.0001	1.63	<0.0001
	(3.00–3.15)		(1.53–1.75)	
Body Mass Index (kg/m^2^)		<0.0001		<0.0001
18.50–24.99	1.00	-	1.00	-
<18.50	0.21	<0.0001	0.25	0.0023
	(0.15–0.29)		(0.11–0.61)	
25.00–29.99	6.64	<0.0001	4.98	<0.0001
	(6.43–6.86)		(4.56–5.44)	
30.00–34.99	31.38	<0.0001	23.93	<0.0001
	(30.32–32.47)		(21.89–26.16)	
35.00–39.99	67.14	<0.0001	55.53	<0.0001
	(63.95–70.48)		(49.54–62.24)	
≥40.00	70.96	<0.0001	67.29	<0.0001
	(65.3–77.10)		(56.39–80.30)	
Blue collar [White collar]	1.48	<0.0001	1.04	0.1680
	(1.45–1.51)		(0.98–1.10)	
Smoking status		<0.0001		<0.0001
Never smoker	1.00	-	1.00	-
Former smoker (stopped smoking ≥1 year ago)	2.19	<0.0001	1.13	0.0008
	(2.14–2.25)		(1.05–1.22)	
Former smoker (stopped smoking <1 year ago)	1.61	<0.0001	1.34	0.0002
	(1.54–1.70)		(1.15–1.56)	
Current smoker	1.34	<0.0001	1.33	<0.0001
	(1.32–1.37)		(1.26–1.41)	
Heavy drinker [Not a heavy drinker]	1.66	<0.0001	1.27	0.0024
	(1.58–1.75)		(1.09–1.48)	
With hypercholesterolemia [Without hypercholesterolemia]	2.92	<0.0001	1.77	<0.0001
	(2.87–2.98)		(1.68–1.86)	
Physical exercise		<0.0001		<0.0001
Regular physical exercise	1.00	-	1.00	-
≥2 h/week of physical exercise	1.61	<0.0001	1.25	<0.0001
	(1.47–1.75)		(1.13–1.38)	
<2 h/week physical exercise	1.46	<0.0001	1.19	0.0001
	(1.35–1.58)		(1.09–1.30)	
No physical exercise	2.05	<0.0001	1.42	<0.0001
	(1.93–2.18)		(1.32–1.52)	

^a^OR: odds ratio

^b^95 % CI: 95 % confidence interval

Reference categories for binary outcomes are included within square brackets

Because physical exercise was recorded only in one fourth of the study participants (*n* = 100,561), we performed another regression analysis excluding this factor. The results of this analysis were almost identical to those reported for the full model (data not shown).

## Discussion

The prevalence of obesity (15.5 %) and overweight (38 %) in our sample was similar to that reported for Spanish young adults (18–44 years) in the general population (15 and 33.4 %, respectively) [15]. The prevalence of the MHO phenotype (8.6 %) in our study, in a working population and using the modified NCEP-ATPIII criteria, is within the range reported in the literature (2–12 %) for the general population [5]; however, it was higher than that reported for the general population in the ENRICA study in Spain (6.5 %) [16]. Subjects from the ENRICA study were older, and more importantly, they were considered metabolically healthy if they had fewer than 2 cardio-metabolic abnormalities [16]. These differences may explain the lower prevalence of MHO in the ENRICA study compared with our results. In our study, even among the most obese subjects, the prevalence of metabolic health was relatively high. Thus, approximately 40 % of individuals in the obese II and III categories were considered to be metabolically healthy. Certainly, these results are influenced by which definition of metabolic health is used. When we used the stricter criteria of not having any criterion of metabolic syndrome, none of these individuals were metabolically healthy. A detailed discussion of the definition of metabolically healthy obesity and its implications can be found elsewhere [5]. We are not aware of other studies on prevalence of the MHO phenotype conducted in the working population.

According to our regression analysis, the factors associated with a metabolically unhealthy phenotype were BMI, age, presence of hypercholesterolemia, male sex, being a smoker or heavy drinker, and undertaking no physical exercise. There are limited data available on the determinants of metabolic health status [5]. Our results in this regard are not fully consistent with those reported by Lopez-García et al. in a representative sample of the Spanish general population [16]. Although the role of age, sex and physical exercise was the same as in our study, they found that the likelihood of being metabolically healthy (that is, having 0–1 cardio-metabolic abnormalities) in obese individuals was higher in current smokers. Among normal weight individuals, the likelihood of being metabolically unhealthy was lower in former and current smokers. Regarding alcohol consumption, the categories used in both studies are not equivalent and, therefore, it is difficult to make any comparison. In our study we found that, for any of the BMI categories, individuals that consumed 14 or more standard drinks per week (heavy drinkers) were more likely to be metabolically unhealthy. Wildman et al., in a representative sample of the US non-institutionalized population, after adjusting for waist circumference, found that among overweight and obese individuals, younger age, non-Hispanic Black race/ethnicity, moderate physical exercise levels and smaller waist circumference were independently associated with a metabolically healthy phenotype [17]. Our findings and those of Wildman et al. [17] suggest the usefulness of targeting modifiable factors with lifestyle interventions. MHO seems to be a transient status [5, 18]; therefore, a sound approach would be to identify metabolically healthy overweight or obese individuals and to initiate lifestyle interventions to avoid their progression to an unhealthy phenotype. The results of a recent prospective study conducted in Spain support this approach [19]. These authors found that a healthy lifestyle (measured with an index that combined diet quality, physical exercise, and smoking status) was associated with a significant lower likelihood of transition to a metabolically abnormal overweight/obese phenotype [19].

Apart from the frequency of hypertension, the most common metabolic risk factor among metabolically unhealthy underweight or normal weight individuals, when compared with those who were metabolically healthy, was an increased level of triglycerides and a greater mean waist circumference. This phenotype overlaps the so-called hypertriglyceridemic waist phenotype. The presence of this phenotype has been associated with subclinical atherosclerosis [20], incident diabetes [21, 22], and an increased risk of coronary artery disease [23, 24]. In fact, some authors have reported that evaluating the presence of the hypertriglyceridemic waist phenotype is as discriminant as the NCEP-ATP III or the International Diabetes Federation criteria for identifying individuals at increased cardio-metabolic risk [25]. Although we have not analyzed the concordance between the hypertriglyceridemic waist phenotype and the metabolically unhealthy phenotype, our results appear to support the usefulness of the hypertriglyceridemic-waist phenotype for identifying individuals who are metabolically unhealthy.

In underweight and normal weight individuals who were metabolically unhealthy, the proportion of blue-collar workers was 66.7 and 72.8 %, respectively, and among those with the healthy phenotype, the corresponding figures were 54.7 and 60.4 %, respectively. Albeit speculative, these differences may be related to differences in lifestyle characteristics. In a study conducted in Finland, unskilled blue-collar workers had more cardiovascular risk factors (namely, smoking, hypertension, and reduced physical exercise during leisure time) than white-collar workers [26].

The high proportion of obese subjects who are metabolically healthy also suggests that BMI is not sufficient as a marker of cardio-metabolic risk and, therefore, that there is a need for the development and validation of other markers that may help to guide treatment-decision making [27].

Our study has several limitations. Its cross-sectional design does not allow us to establish causal relationships. In fact, when evaluating the factors associated with the metabolically unhealthy phenotype, the direction of the association was assumed to be that evaluated in the model. In our study, there is a lack of information regarding some other variables that have been used to define metabolic health status such as the homeostatic model assessment of insulin resistance (HOMA-IR). However, it should be noted that there is not a standardized cut-off value of HOMA-IR to define metabolic health [18]. The strengths of this study include the sample size, the study setting, using a sample that is representative of the Spanish working population, and the analysis of a subpopulation that has scarcely been investigated, namely underweight individuals.

## Conclusions

Our study shows that prevalence of MHO individuals in a large sample of the working population is high, corresponding to more than half of the obese individuals. The factors associated with the presence of the metabolically unhealthy phenotype include several modifiable risk factors such as relative weight, smoking, heavy drinking, and no physical exercise. Detecting, at an early stage, obese and overweight individuals who are metabolically healthy may be useful to reduce the likelihood of transition to a metabolically unhealthy phenotype by allowing targeting of the aforementioned risk factors with lifestyle modification initiatives. The working environment seems an appropriate setting to implement those initiatives in conjunction with the Public Health Services.

## Abbreviations

BMI: body mass index
CI: confidence interval
HDL: high density lipoprotein
HOMA-IR: homeostatic model assessment of insulin resistance
ICARIA: Ibermutuamur Cardiovascular Risk Assessment
MHO: metabolically healthy obese
MUHO: metabolically unhealthy obese
NCEP-ATPII: National Cholesterol Education Program Adult Treatment Panel III
OR: odds ratio
